# Exercise Testing in Children and Adolescents With Hypertrophic Cardiomyopathy: Let’s Get Moving!∗

**DOI:** 10.1016/j.jacadv.2022.100111

**Published:** 2022-10-05

**Authors:** Michael Khoury, Jennifer Conway

**Affiliations:** Department of Pediatrics, University of Alberta, Edmonton, Alberta, Canada

**Keywords:** exercise, hypertrophic cardiomyopathy, pediatric

**Figure:**
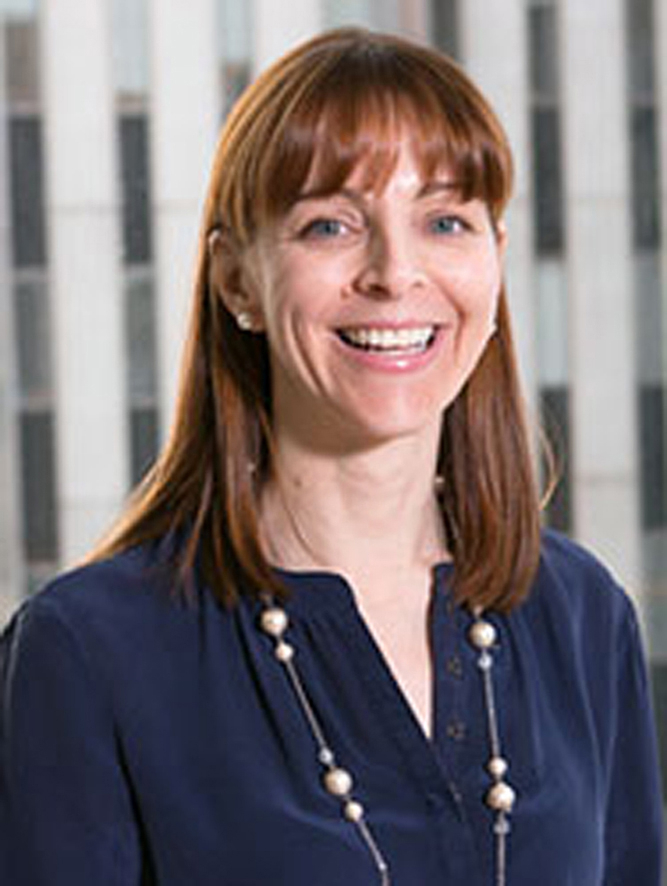

Hypertrophic cardiomyopathy (HCM) exists along a wide clinical spectrum in children and adolescents. While most children can expect a relatively benign course throughout childhood and into adulthood, childhood is associated with a relatively higher risk of life-threatening cardiac arrest events.^1^ Moreover, some children and adolescents experience progression of disease severity, including the development of progressive and dynamic left ventricular outflow tract (LVOT) obstruction and advanced heart failure symptoms. Accurately risk stratifying children to predict the occurrence of short- and long-term HCM complications continues to be a significant challenge.

Exercise stress testing (EST) is a useful modality to evaluate the safety of exercise and inform the development of personalized exercise recommendations and interventions in children with congenital and acquired heart disease. It allows for a dynamic evaluation of a patient’s disease severity unlike more routine evaluations which are typically performed at rest and are limited in their ability to predict real-world anatomic and physiologic adaptations to stress. Traditionally, hesitancy has existed with respect to the safety of exercise in children with HCM, with prior guidelines recommending lower intensity and less dynamic competitive and recreational sport and physical activity for most patients.^2^ This has perhaps inadvertently extended to safety concerns around EST, resulting in its relative underutilization, despite the most contemporary guidelines recommending its incorporation into the routine evaluation for children and adults with HCM.^1^ While EST has been increasingly utilized in the care of adults with HCM,^3^ the pediatric experience is relatively underdescribed.

In this issue of *JACC: Advances*, Edelson et al^4^ sought to evaluate the safety and utility of EST in children with HCM, specifically evaluating its role in predicting future major cardiovascular events. To do so, they undertook an ambitious single-center retrospective review over the span of nearly 2 decades. Children and adolescents younger than 21 years who underwent EST at the Children’s Hospital of Philadelphia between 2000 and 2018 (inclusive) were studied. A primary composite endpoint was utilized, including death, aborted cardiac death, transplant, or arrhythmia prompting implantable cardioverter-defibrillator (ICD) placement. There were 140 patients included. Of these, only 2 required test abortion due to safety concerns. The median exercise capacity was in the low-normal range at 81% predicted (37.1, IQR: 12.5 ml/kg/min). Curiously, 80% of the included patients were male. Given the autosomal dominant nature of HCM, the reasoning behind the relative underrepresentation of females in the study is not immediately clear and requires further consideration regarding clinical practice patterns. Notably, 8% of the patients had an abnormal blood pressure response (ABPR) to exercise, and 44% had some element of atrial or ventricular ectopy at some point during the test, including when at rest or in recovery following the test.

The composite outcome occurred in about 9% of patients (12/140). Despite being very broadly defined, the occurrence of any form of ectopy was the only clinical variable associated with the composite outcome, with 10 of the 12 patients who had the composite outcome having some form of ectopy during their EST. Taking a more granular approach to these patients reveals a broad spectrum of ectopy severity during the EST, ranging from isolated atrial ectopy in recovery to monomorphic ventricular tachycardia during peak exercise. These findings suggest that the presence of any form of ectopy during EST may be a sensitive measure for future risk of major adverse cardiac events in the pediatric HCM population. The utility of this potential risk factor requires further exploration through larger, prospective analyses. Conversely, none of the patients who experienced the composite outcome had an ABPR to exercise, a notable finding as this has been previously identified as a risk factor for death in HCM for children.^5^ Of note, ABPR was previously incorporated as a risk factor for primary prevention placement of ICD in previous guidelines,^2^ and in the most updated guideline, it has been removed^1^ and is not included in either risk scores recently developed for pediatric populations.^6^^,^^7^

While the findings in this work are reassuring with respect to the safety of EST in pediatric patients with HCM, it is important to recognize the potential for selection bias. We simply do not know the safety of EST for children with HCM who were not sent for testing. Such children may have had a more severe phenotype of HCM. Supporting this notion was the finding that the median resting LVOT gradient of included patients was 0 mm Hg (IQR: 24 mm Hg). In a separate single-center experience of exercise stress echocardiography, a higher-risk cohort was included, with baseline obstruction in 29% and 29% having prior ICD implantation prior to their EST.^8^ One of these patients had a cardiac arrest during his EST due to the occurrence of fast polymorphic ventricular fibrillation, with successful resuscitation including 2 ICD shocks and 2 external shocks. The true safety of EST (and by extension, physical activity and recreational and competitive sports participation) must be considered on an individualized basis, particularly for patients with more severe phenotypes of disease. Decisions in the face of uncertainty should thus incorporate shared decision-making with the patient and their family, as recommended in current guidelines.^1^

As suggested by the authors, the relative rarity of the composite outcome occurrence, when combined with the limited sample size obtained from a single-center retrospective review, limits the statistical power to further evaluate demographic and clinical features predictive of future major cardiovascular events. Larger prospective multicenter studies through established networks are needed for more thorough evaluations. For example, the authors of the present study were unable to evaluate the clinical implications of changes in exercise capacity over time across sequential ESTs. Rather, they studied the most recent EST within the study period or prior to the occurrence of the composite endpoint. Evaluating the clinical implications of various trajectories in exercise capacity over time would further highlight the prognostic utility of EST in the HCM population. Such associations have been previously demonstrated in other high-risk populations, including those with a Fontan circulation.^9^ The study did, however, demonstrate a decline in exercise capacity with age in the present study, a finding that has been demonstrated to occur in other pediatric cardiac populations to a greater degree than for those without heart disease.^10^ The mechanisms surrounding this require further evaluation but may be due to chronic physical inactivity and advancing disease severity through adolescence.

The study authors are to be commended on the routine use of EST in children with HCM at their center, in keeping with the most contemporary guidelines.^1^ The broader experience across pediatric centers in the United States and internationally with EST in this population is not well described. Moreover, the incremental utility of exercise stress echocardiography over conventional EST to demonstrate provocable LVOT obstruction has shown promise in pediatric HCM patients but requires further study.^8^ Exercise stress echocardiography has been studied to a greater degree in the adult HCM population and has demonstrated utility in evaluating need for medical and surgical therapy and response to therapy, while also being a useful prognostic tool.^3^

EST is a useful, albeit likely underutilized, tool with potential prognostic implications for children and adolescents with HCM, particularly when echocardiography is incorporated. While it appears to be safe in certain patients, the external validity of EST in this patient population is limited in part by the single-center study designs to date with the potential for selection bias. Current established networks should now aim to study and implement routine EST assessments in order to fully realize the potential of this test in predicting outcomes and to inform safe and effective exercise strategies for children and adolescents with HCM.

## Funding support and author disclosures

The authors have reported that they have no relationships relevant to the contents of this paper to disclose.
